# Endovascular treatment for traumatic thoracic aortic pseudoaneurysm: a case report

**DOI:** 10.1186/1749-8090-8-36

**Published:** 2013-03-03

**Authors:** Po-Sung Li, Chung-Lin Tsai, Tzu-Chieh Lin, Siu-Wan Hung, Sung-Yuan Hu

**Affiliations:** 1 Department of Emergency Medicine, Taichung Veterans General Hospital, Taichung, Taiwan; 2 Division of Cardiovascular Surgery, Department of Surgery, Taichung Veterans General Hospital, Taichung, Taiwan; 3 Department of Radiology, Taichung Veterans General Hospital, Taichung, Taiwan; 4 School of Medicine, Chung Shan Medical University, Taichung, Taiwan; 5 School of Medical Imaging & Radiological Sciences, Chung Shan Medical University, Taichung, Taiwan; 6 Institute of Medicine, Chung Shan Medical University, Taichung, Taiwan; 7 Department of Nursing, College of Health, National Taichung University of Science and Technology, Taichung; 8 Department of Veterinary Medicine, National Chung Hsing University, Taichung, Taiwan

**Keywords:** Computed tomographic angiography (CTA), Endovascular stent-graft, Thoracic aortic pseudoaneurysm (TAPA), Traumatic aortic injury (TAI)

## Abstract

Cases of an endovascular treatment for traumatic aortic injury are extremely rare. A prompt diagnosis of traumatic thoracic aortic pseudoaneurysm through a 3-dimensional computed tomographic angiography of aorta and emergency repair are mandatory to rescue the life-threatening condition. An endovascular treatment is a trend for traumatic aortic injury because of lower invasivity, morbidity and mortality. We reported a rare case of traumatic aortic injury with thoracic aortic pseudoaneurysm definitively diagnosed by the reconstructional computed tomographic angiography of aorta and successfully treated with endovascular stent-graft.

## Background

Traumatic aortic injury has been associated with a lethal surgical lesion and a high mortality in blunt chest trauma. The risk of aortic rupture in traumatic aortic injury has been reported approximately 5% in the acute phase [1]. Thoracic aortic pseudoaneurysm is a rare and life-threatening complication of traumatic aortic injury with an incidence of 2%–5% [2]. The most commonly involved site is isthmus. 93%–98% of patients with traumatic aortic injury have been a definitive diagnosis by computed tomographic scan and thoracic aortic pseudoaneurysm in computed tomographic scan is a direct sign of traumatic aortic injury [1-3]. In hemodynamic unstable patients of traumatic aortic injury, an endovascular treatment with stent-graft offers a suitable alternative to open surgical intervention [1,4-8].

## Case presentation

**Figure 1:**
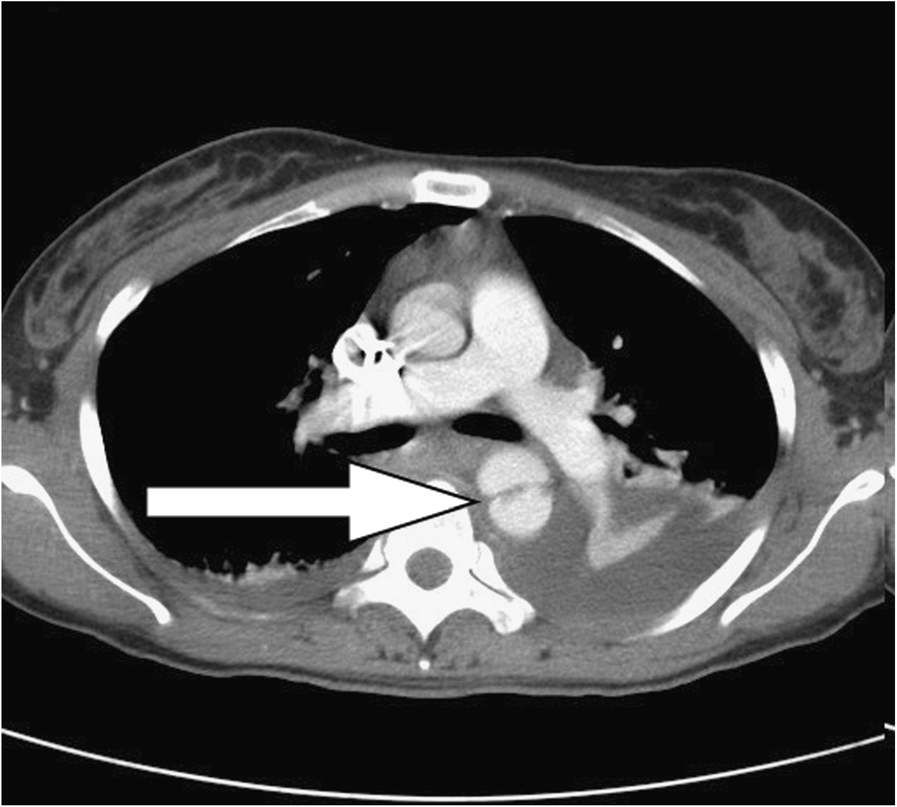
The axial view of computed tomographic angiography (CTA) revealed a dumbbell-shaped aorta (arrow) with hemothorax and hemomediastinum consistent with a ruptured thoracic aortic pseudoaneurysm.

**Figure 2:**
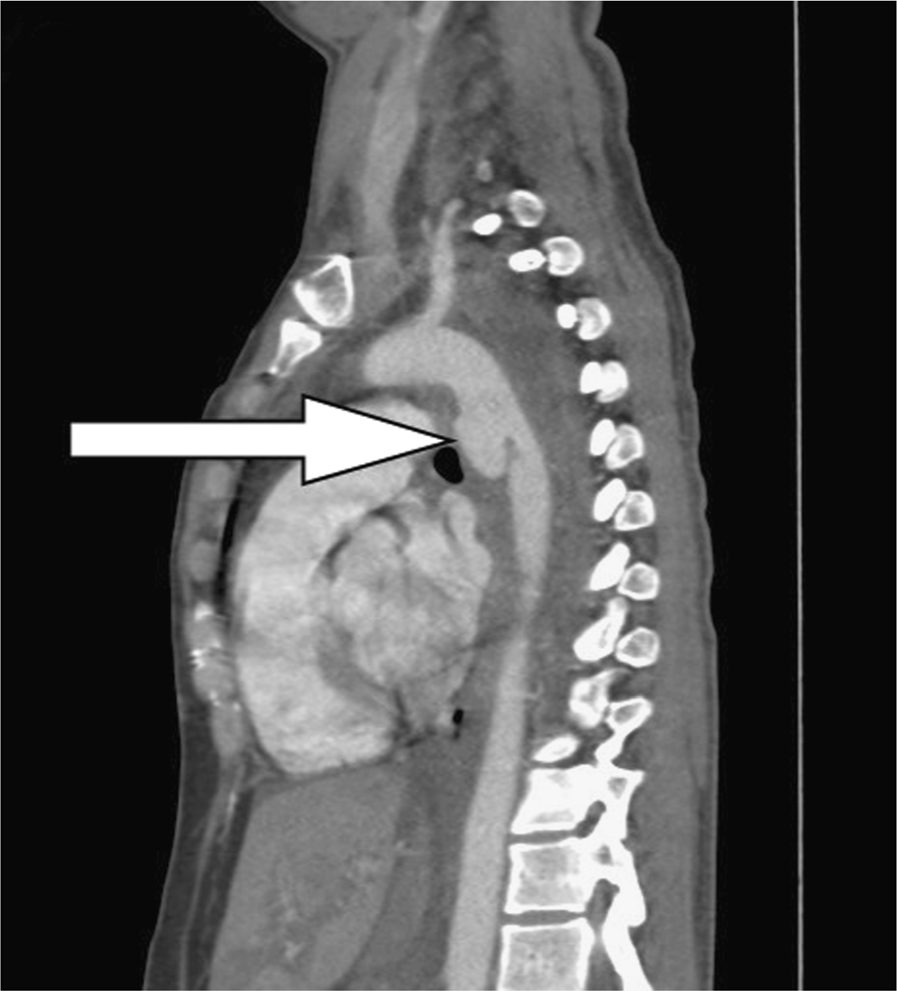
The coronal view of CTA depicted a thoracic aortic pseudoaneurysm (arrow) below the aortic arch, hemothorax and hemomediastinum.

**Figure 3:**
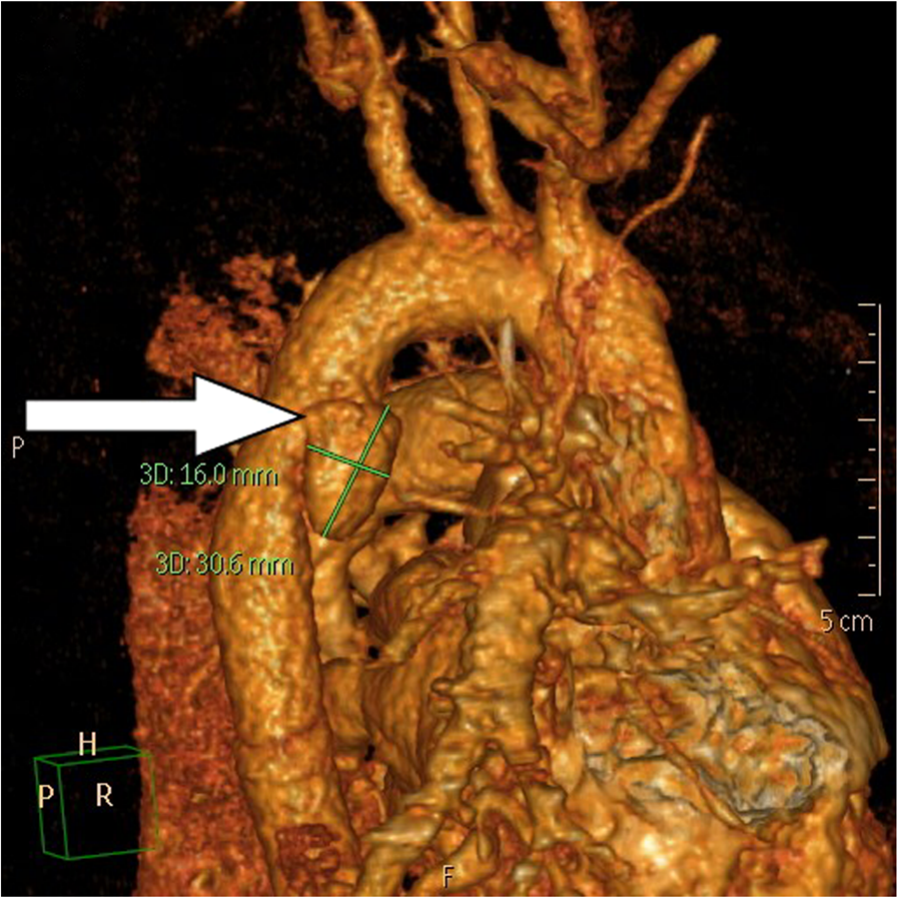
The reconstructional image of CTA demonstrated a thoracic aortic pseudoaneurysm of 16 mm × 30.6 mm (arrow) in size.

**Figure 4:**
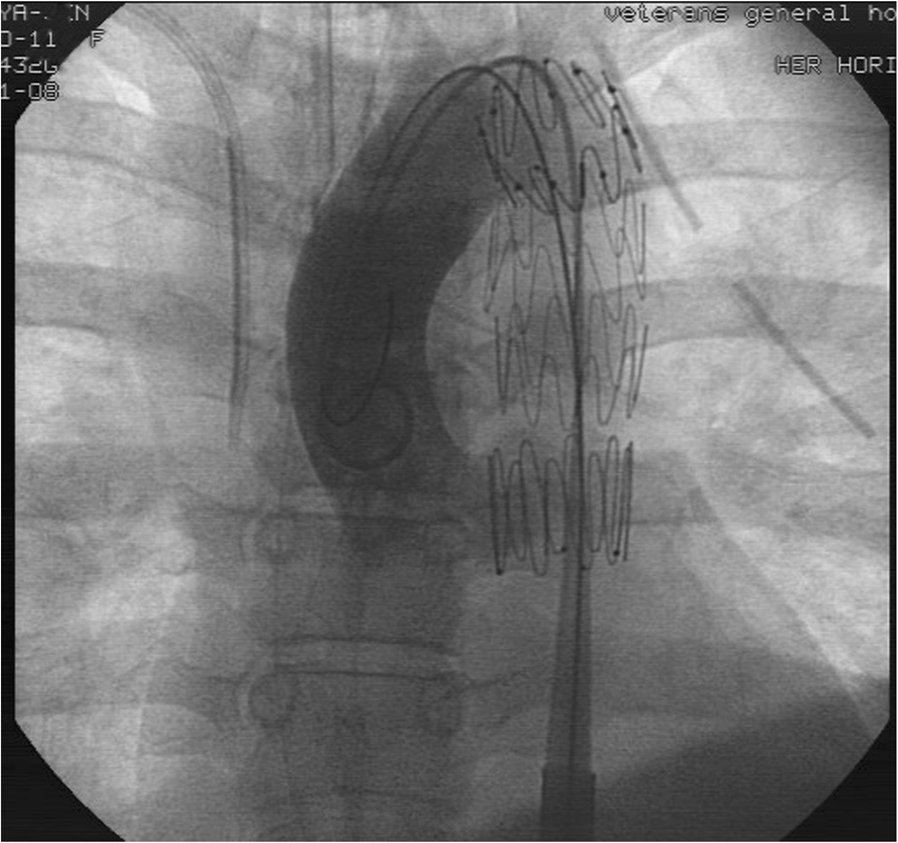
**An emergency aortography and an endovascular repair with a fenestrated stent-graft (COOK®, Zenith®TX2®TAA Endovascular Graft, Proximal Extensions, Order number: TBE-26-80) were conducted successfully for the thoracic aortic pseudoaneurysm.**

A 28-year-old healthy unmarried woman committed suicide with a high-rise fall of 20 meters and presented hypovolemic shock caused by blunt thoracic, abdominal injury and left femoral shaft fracture to our emergency department. Upon admission, vital signs were respiratory rate of 22/min, blood pressure of 88/40 mmHg, pulse rate of 106/min, and body temperature of 33°C. Physical examination showed a 5 cm-laceration wound over submandible, a Glasgow Coma Scale of E1V2M1, rapid regular heart beats, absent breathing sound of left lung filed, tenderness over upper abdominal region and deformity with tenderness of left thigh. Standard advanced trauma life support with an endotracheal intubation for protection of airway, fluid resuscitation with normal saline and blood transfusion for unstable hemodynamic status and essential survey of images was conducted. Laboratory investigations were white blood cell counts of 9,300/mm^3^, hemoglobin of 11.3 g/dl, platelet counts of 287 × 10^3^/mm^3^, blood urea nitrogen of 15 mg/dl, creatinine of 1.0 mg/dl, sodium 137 mEq/l, potassium 3.4 mEq/l, calcium 7.4 g/dl, albumin 3.3 of g/dl, total protein of 6.0 g/dl, glutamic-oxalacetic transaminase of 711 U/l, glutamic-pyruvic transaminase of 302 U/l, lactic dehydrogenase of 1002 U/l, creatine kinase of 485 U/l, and troponin-I of < 0.034 ng/ml. Arterial blood gas analysis was a pH of 7.190, P_a_O_2_ of 85.5 mmHg, P_a_CO_2_ of 39.2 mmHg, and HCO3^-^ of 14.6 mmol/l. Chest plain film demonstrated a fracture of left first rib, abnormal contour of aortic arch with hemothorax and patchy infiltration at right lower lung suggestive of aortic injury and lung contusion (Additional file 1: Figure S1). Plain film of left femur showed a fracture of femoral shaft (Additional file 1: Figure S2). The reconstructional images through contrast-enhanced computed tomographic angiography (CTA) of aorta demonstrated a thoracic aortic pseudoaneurysm (TAPA) of 16 mm × 30.6 mm (Figures 1, 2 and 3) with hemothorax, hemomediastinum and liver laceration of left lobe with hemoperitoneum (Additional file 1: Figure S3). Six hours later, an endovascular treatment with stent-graft (Figure 4) was performed successfully. Open reduction with internal fixation for fracture of left femoral shaft was performed 6 days later. She was ultimately discharged on the 18th postoperative day after an uneventful admission.

## Discussion

Chest trauma is classified as blunt or penetrating and the former is the most cause of thoracic injuries with an incidence of 90%. The prevalence of thoracic injuries caused by falls from heights is 10–20%. Traumatic aortic rupture is a very rare condition with an average of 2.2 cases per center per year reported in North America. Thoracic great vessel related to blunt trauma is relatively rare with an incidence of less than 5% [5-8].

The mechanism is torsion and shearing forces against the aorta at levels of relative immobility, mainly the ligamentum arteriosum with an incidence of 90–95%. The isthmus, a part of relatively mobile thoracic aorta joining the fixed arch and the insertion of the ligamentum arteriosus, becomes the most commonly injured site in deceleration accident, with an involved incidence of 80% and 90–95% in the pathological and clinical series, respectively [1-8].

The traumatic aortic injury (TAI), a lesion extending from the intima to the adventitia, usually occurs in deceleration accidents, such as motor vehicle collisions, falls from height or crush injuries, with an immediately high mortality rate of 80–90% during the first phase, so the victims should be taken to hospital as quickly as possible. Survival patients in the initial phase of TAI have a higher successful outcome under endovascular repair comparative of conventional open surgical repair [1-8]. The predictors of TAI include age older than 50 years, being unrestrained, systolic blood pressure of less than 90 mmHg, thoracic injury, abdominopelvic injury with fractures of the lumbar spine and pelvis, long bone fractures, and major head injury [7].

Widening of mediastinum greater than 8 cm and/or 25% of the width of the thorax, loss of aortico-pulmonary window, tracheal deviation to the right, nasogastric shifting to right, left apical cap, depression of the left main stem bronchus, left-sided hemothorax, or scapular, sternal, thoracic spine or multiple rib fractures on plain radiography is suggestive of aortic rupture [5,7,9]. Fractures of the first and second ribs are clearly markers of severe blunt force trauma [7,9].

Multidetector CTA is the diagnostic modality for the initial evaluation and accurate diagnosis of TAI, with a sensitivity of 98% and a specificity of 100% [6,7]. CTA can offer a non-invasive assessment of the anatomical characteristics of TAI with rapid deceleration force or clinical suspicion. A prompt diagnosis of traumatic TAPA through a 3-dimensional CTA of aorta and emergency repair with stent graft are mandatory to rescue the life-threatening condition. An endovascular repair is a trend with greater and greater acceptance for TAI because of lower invasivity, avoiding thoracotomy and use of heparin, lower morbidity and mortality compared with conventional open surgical repair [1-10].

Pseudoaneurysm formation, intraluminal filling defect, and intimal irregularity are common findings in vessel wall injury [7]. Direct signs of TAI include active contrast medium extravasation, an intimal flap, TAPA, an increase in size of the periaortic hematoma, aortic contour/diameter variation, contained rupture, intraluminal mural thrombus, abnormal aortic contour, recurrent hemothorax and abrupt change in aortic caliber (pseudocoarctation) [1-3,6,7]. Indirect CT signs are indistinctness of mediastinal flat planes, periaortic haematoma and mediastinal haematoma [2,3,6].

Short-term complications of endovascular repair for TAI include paraplegia, stroke, puncture-site complications, device collapse, endoleak and recurrent laryngeal nerve damage. However, very little data are available concerning long-term outcomes and complications [5,7]. We highlight that CTA and emergency endovascular repair of stent-graft for TAI with TAPA in polytrauma patients are recommended.

## Conclusion

Prompt diagnosis and management of highly suspicious patients with traumatic thoracic aortic pseudoaneurysm caused by blunt chest injury is associated with the good prognosis. We provide the successful experience of a less invasive intervention with an endovascular stent-graft for traumatic thoracic aortic pseudoaneurysm through the mechanism of rapid deceleration.

## Consent

Written informed consent was obtained from the patient for publication of this case report and accompanying images. A copy of the written consent is available for review by the Editor-in-Chief of this journal.

## Competing interests

The authors declare that they have no competing interests.

## Authors’ contributions

LPS, HSY and LTC conducted primary survey, resuscitation and emergency care at emergency department. HSW prepared the radiological images. TCL performed the endovascular repair. LPS and HSY participated in the design of the case report and performed the search in the literature. LPS, HSY, LTC, HSW and TCL participated in the design and coordination of the report. All authors read and approved the final manuscript.

## Supplementary Material

Additional file 1: Figure S1 Chest x-ray revealed fracture of left first rib (arrow) and a widening of left upper mediastinum with a pattern of ground glass of left lung, an abnormal contour of aortic arch, loss of aortopulmonary window and an infiltrative patchy lesion over right lower lung suggestive of aortic injury and lung contusion. **Figure S2.** The plain film of radiography showed a fracture of left femoral shaft. Open reduction with internal fixation for fracture of left femoral shaft was performed 6 days later. **Figure S3.** Computed tomographic angiography demonstrated liver laceration of left lobe with hemoperitoneum under conservative treatment. Click here for file
