# Supplementary material for: Endovascular treatment for traumatic thoracic aortic pseudoaneurysm: a case report
Source: J Cardiothorac Surg. 2013 Mar 3;8:36. doi: 10.1186/1749-8090-8-36 (PMC3639143; doi:10.1186/1749-8090-8-36)

Figure S1: Chest x-ray revealed fracture of left first rib (arrow) and a widening of left upper mediastinum with a pattern of ground glass of left lung, an abnormal contour of aortic arch, loss of aortopulmonary window and an infiltrative patchy lesion over right lower lung suggestive of aortic injury and lung contusion.


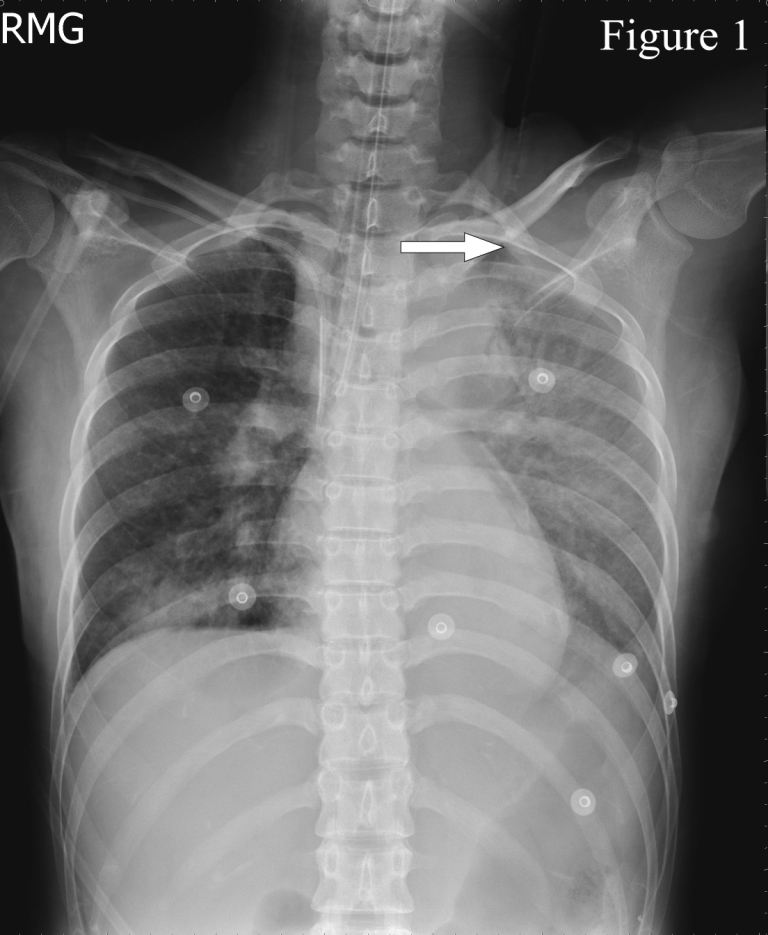


Figure S2: The plain film of radiography showed a fracture of left femoral shaft. Open reduction with internal fixation for fracture of left femoral shaft was performed 6 days later.


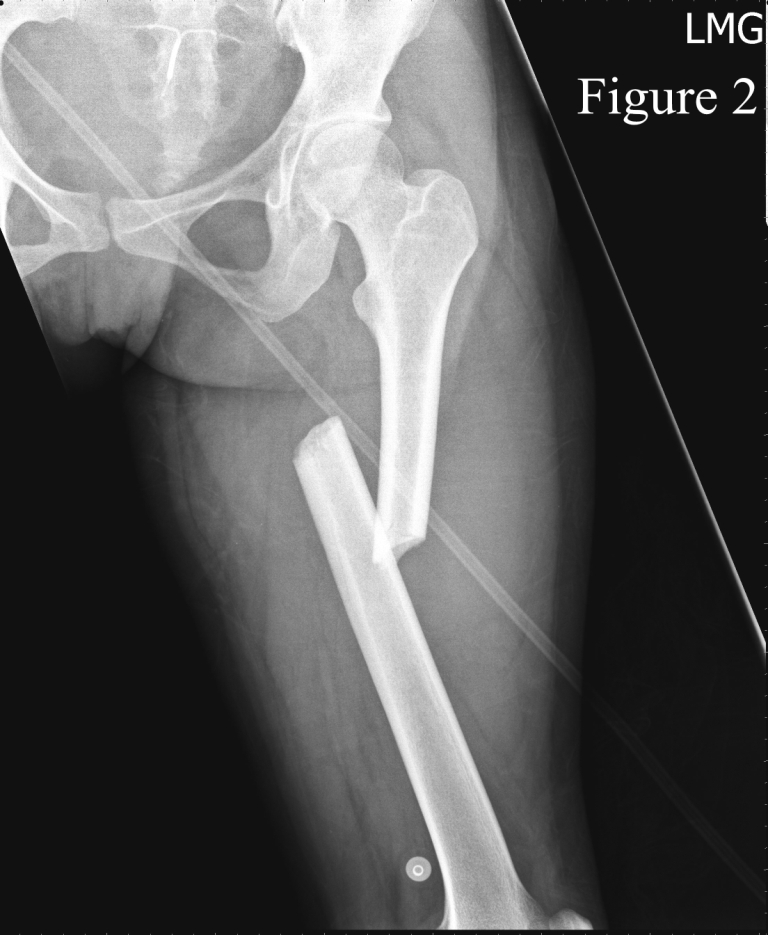


Figure S3: Computed tomographic angiography demonstrated liver laceration of left lobe with hemoperitoneum under conservative treatment.


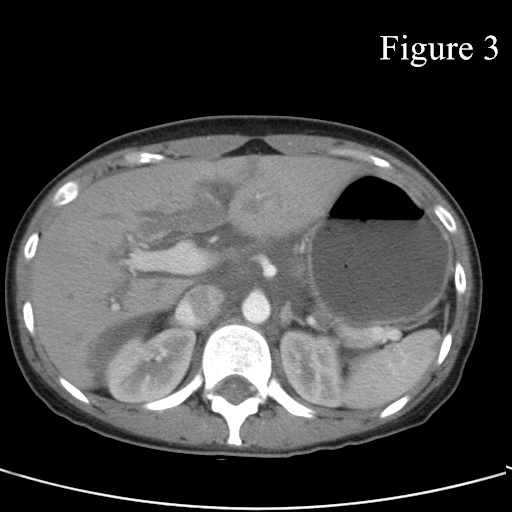

Supplement: Additional file 1: Figure S1 — Chest x-ray revealed fracture of left first rib (arrow) and a widening of left upper mediastinum with a pattern of ground glass of left lung, an abnormal contour of aortic arch, loss of aortopulmonary window and an infiltrative patchy lesion over right lower lung suggestive of aortic injury and lung contusion. Figure S2. The plain film of radiography showed a fracture of left femoral shaft. Open reduction with internal fixation for fracture of left femoral shaft was performed 6 days later. Figure S3. Computed tomographic angiography demonstrated liver laceration of left lobe with hemoperitoneum under conservative treatment. [file 1749-8090-8-36-S1.doc]
